# Hydrogen Embrittlement of 27Cr−4Mo−2Ni Super Ferritic Stainless Steel

**DOI:** 10.3390/ma17071546

**Published:** 2024-03-28

**Authors:** Fei Yang, Yujin Nie, Huiyun Zhang, Weiqiang Niu, Quanxin Shi, Jinyao Ma, Liuwei Zheng, Wei Liang

**Affiliations:** 1 College of Materials Science and Engineering, Taiyuan University of Technology, Taiyuan 030024, China; yangfei0911@163.com (F.Y.);; 2 Instrumental Analysis Center, Taiyuan University of Technology, Taiyuan 030024, China

**Keywords:** super ferritic stainless steel, hydrogen embrittlement, mechanical properties, yield strength, fracture behavior

## Abstract

The effect of hydrogen content on the deformation and fracture behavior of 27Cr−4Mo−2Ni super ferritic stainless steel (SFSS) was investigated in this study. It was shown that the plasticity and yield strength of SFSS were very susceptible to hydrogen content. The introduction of hydrogen led to a significant decrease in elongation and a concurrent increase in yield strength. Nevertheless, a critical threshold was identified in the elongation reduction, after which the elongation remained approximately constant even with more hydrogen introduced, while the yield strength exhibited a monotonic increase with increasing hydrogen content within the experimental range, attributed to the pinning effect of the hydrogen Cottrell atmosphere on dislocations. Furthermore, the hydrogen-charged SFSS shows an apparent drop in flow stress after upper yielding and a reduced work hardening rate during the subsequent plastic deformation. The more hydrogen is charged, the more the flow stress drops, and the lower the work hardening rate becomes.

## 1. Introduction

Super ferritic stainless steel (SFSS) is extensively utilized in heat exchange equipment using various water sources as a cooling medium, including seawater, because of its excellent corrosion resistance, high thermal conductivity, low coefficient of thermal expansion, low production cost, and nickel savings [1,2,3,4]. Nonetheless, prolonged exposure to harsh environments such as seawater may result in hydrogen embrittlement (HE) [5]. To mitigate corrosion effects, cathodic protection is employed, but this can increase the risk of hydrogen embrittlement. Furthermore, to enhance the resistance of SFSS against pitting and crevice corrosion, substantial amounts of alloying elements such as Cr, Mo, and Ti, among others, are incorporated. This strategy not only enhances corrosion resistance but also markedly boosts the room-temperature yield strength via solid-solution reinforcement [6,7,8], consequently increasing the susceptibility to HE. Therefore, understanding the behavior of HE in SFSS is crucial for its practical applications in engineering.

Hydrogen embrittlement refers to the significant reduction in mechanical properties, such as ductility, toughness, and fatigue life, caused by the introduction of hydrogen atoms in a hydrogen environment [9,10,11]. HE is a multifaceted phenomenon intricately tied to the microstructure of the material as well as the concentration and distribution of hydrogen atoms within the material. Extensive research has been conducted on the hydrogen embrittlement of various types of steel, including austenitic stainless steel [12,13], ferritic stainless steel [14], duplex stainless steel [15], etc. Several hydrogen embrittlement mechanisms have been proposed to explain the behavior of hydrogen embrittlement in steel, such as the hydrogen enhanced decohesion mechanism (HEDE), hydrogen enhanced localized plasticity (HELP), and adsorption-induced dislocation (AIDE). The HEDE mechanism, which was first introduced by Troiano in 1959, suggests that dissolved hydrogen reduces the cohesive strength of the lattice and interfaces [16,17], leading to crack formation and material failure. In the HELP model, hydrogen accumulation near the crack tip decreases the resistance to dislocation motion, thereby increasing the mobility of dislocation [18]. Birnbaum et al. [19] observed that hydrogen increased dislocation movement by in-situ transmission electron microscopy (TEM) and found that hydrogen reduced the microscopic yield stress. Nevertheless, investigations regarding the HE of super-SFSS are scant, leaving the impact of hydrogen content on SFSS fracture behavior and mechanisms shrouded in ambiguity.

In this study, the effect of hydrogen content on the mechanical behavior of 27Cr−4Mo−2Ni super ferritic stainless steel was evaluated via tensile testing on hydrogen-pre-charged SFSS samples.

## 2. Experimental Procedure

An annealed 27Cr−4Mo−2Ni SFSS used in this study was supplied by TISCO Company of China (Taiyuan, China). The steel had a thickness of 0.8 mm, and its chemical composition is detailed in Table 1.

**Figure 1:**
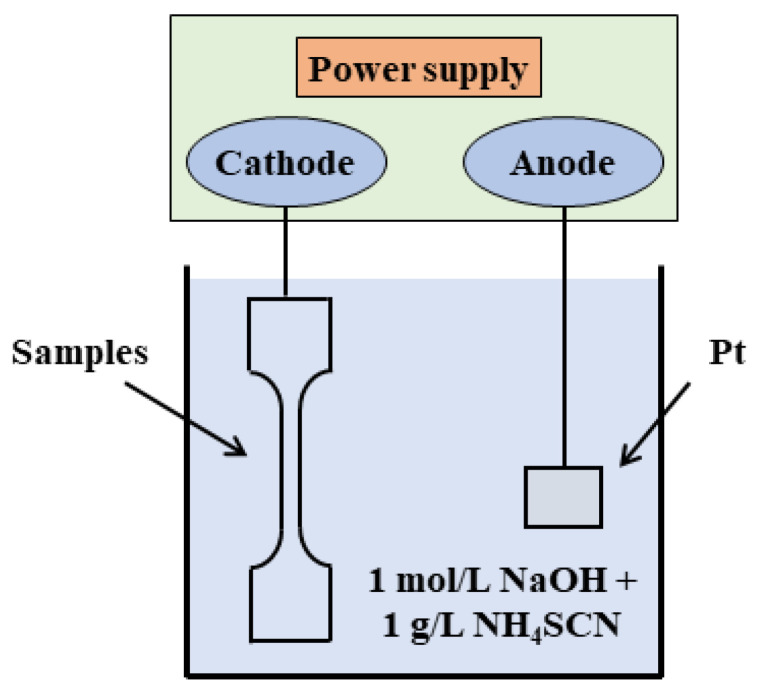
Schematic of the electrochemical cathode hydrogen charging system.

The electrochemical cathode hydrogen charging experiment was performed at room temperature in a 1 mol/L NaOH solution supplemented with 1 g/L thiourea, employing a current density of 50 mA/cm^2^ to introduce hydrogen into the samples. The samples served as the cathode, while a platinum sheet functioned as an anode; both were fully submerged during the hydrogen charging, as depicted in Figure 1. The electrochemical reaction [20] is as follows:

$$H^{+}\left(aq\right)+e^{-}↔\left(\frac{1}{2}\right)\;H_{2}\;\left(g\right)↔H\;\left(chem.\right)$$

NH_4_SCN is incorporated to impede the amalgamation of hydrogen atoms. Electrochemical cathode hydrogen charging is sensitive to the surface quality of the samples, so all the samples underwent grinding with SiC papers ranging from 500 to 3000 grit, followed by polishing before the hydrogen charging procedure.

Thermal desorption spectroscopy (TDS) utilizing JTF-20A equipment was employed to quantitatively measure the cumulative desorbed hydrogen and identify the hydrogen trapping sites. TDS experiments were performed under a continuous flow of Ar gas ranging from 30 °C to 800 °C, with specific heating rates (100, 200, and 300 °C/h used in this study) to obtain the hydrogen desorption profiles. The dimensions of the TDS samples were approximately 45 mm × 6 mm × 0.8 mm. All samples after hydrogen charging were subsequently cleaned with distilled water and ethanol and dried. To prevent the escape of diffusible hydrogen, the TDS measurements started within five minutes after electrochemical hydrogenation.

**Figure 2:**
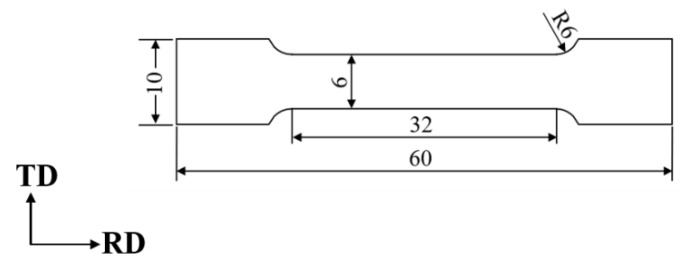
Dimension of samples for tensile (in mm).

The tensile experiments were performed using an electronic material testing machine, employing a strain rate of 3.3 × 10^−4^ s^−1^ at room temperature. Tensile specimens were extracted from the original plate in alignment with the rolling direction, with sample dimensions outlined in Figure 2. The diffusivity of hydrogen in the BCC structure is relatively high even at room temperature, with a hydrogen diffusion coefficient reaching 10^−5^ cm^2^/s [21,22]. To mitigate the impact of hydrogen atoms diffusing out of the samples and affecting the experimental outcomes, the time between the end of electrochemical hydrogen charging and the beginning of stretching was controlled within 10 min. To ensure the accuracy of the experimental results, all the tensile experiments were conducted three times.

In order to quantitatively describe tensile ductility, the hydrogen embrittlement sensitivity index $I_{HE}$ is calculated by the following equation [23] (1):

(1) $$I_{HE}\left(\%\right)=\frac{\delta_{0}-\delta_{H}}{\delta_{0}}\times 100$$

where $\delta_{0}$ and $\delta_{H}$ are the total elongation obtained after tensile testing of the pristine and the hydrogen-pre-charged samples, respectively.

The fracture morphology of all the tensile specimens was studied by ultra-high field emission scanning electron microscopy (JSM 7900F, JEOL, Tokyo, Japan). The microstructure of the pristine sheet and the tensile fracture were characterized by high-resolution scanning electron microscopy (HRSEM, S8000, TESCAN, Brno, Czech Republic) equipped with electron backscatter diffraction (EBSD, Oxford, UK). EBSD images were observed under the following specific conditions: landing energy of 20 kV and a beam current of 3 nA. We were using the Channel 5 software (version 5.12.74.0) to collect and index Kikuchi patterns. The samples were electrochemically polished at 25 V and −30 °C using a solution of 5 mL HClO_4_ and 95 mL C_2_H_5_OH.

## 3. Results

### 3.1. Hydrogen Content

**Figure 3:**
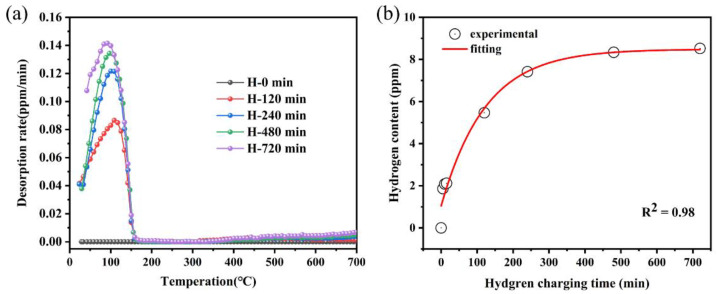
(**a**) The TDS curves of samples hydrogen-charged at various times for 50 mA/cm^2^; (**b**) hydrogen content after charging at different times.

Figure 3a illustrates the hydrogen desorption profiles of 27Cr−4Mo−2Ni SFSS (hydrogen desorption rate vs. heat temperature curves; 100 °C/h heating rate) for different times of hydrogen charge. In Figure 3a, two hydrogen desorption peaks were observed in each hydrogen-pre-charged sample compared with the uncharged sample. Hydrogen liberation occurred within the 30–160 °C temperature range in all hydrogen-pre-charged samples, with a minor release observed upon further heating to 400–600 °C. The intensity of the hydrogen desorption peak value in the curves increased with increasing hydrogen charge time, indicating an increase in the hydrogen content in the samples with longer hydrogen charge duration. Figure 3b demonstrates the hydrogen content in the samples after varying durations of charging at a current density of 50 mA/cm^2^. The diffusion of hydrogen atoms is governed by the concentration gradient, with a steeper gradient resulting in faster hydrogen atom diffusion. As the hydrogen charging time was extended, the hydrogen concentration within the samples initially exhibited a significant increase, followed by a period of more gradual change. After 720 min of hydrogen charging, the concentration in the samples approached saturation. The hydrogen concentration in the pre-charged samples reached a saturation point upon increasing the hydrogen charging duration to 720 min. Despite employing a higher current density for hydrogen charging in this study compared to previous reports [24,25], the trend observed was consistent with the literature, demonstrating that the hydrogen content in the samples will approach saturation after a certain charging period under the given conditions.

### 3.2. Mechanical Properties

**Figure 4:**
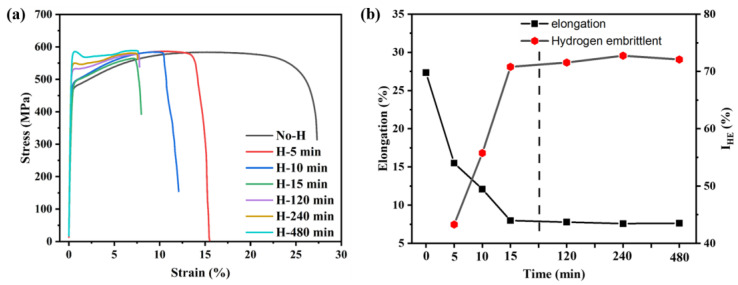
Tensile properties of uncharged and charged samples: (**a**) engineering stress-train curves; and (**b**) elongation and hydrogen embrittlement index *I_HE_*.

**Figure 5:**
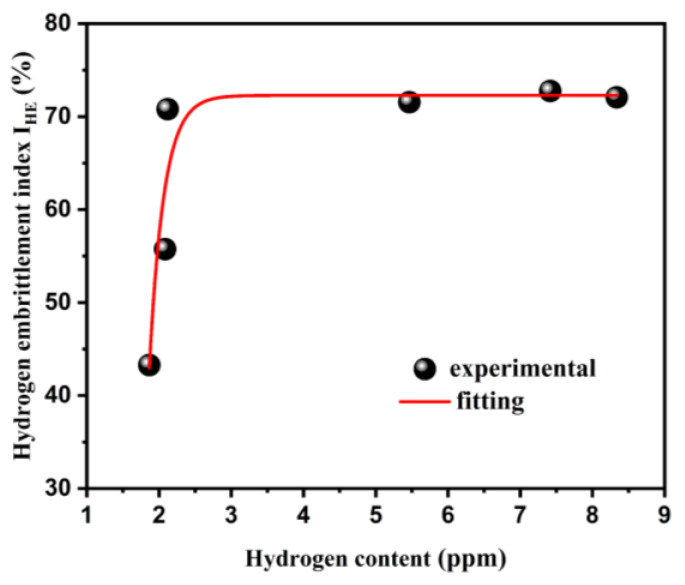
Variation of hydrogen embrittlement sensitivity of the 27Cr−4Mo−2Ni SFSS with hydrogen content.

The tensile test samples were charged with hydrogen in an electrolyte solution for different times under a current density of 50 mA/cm^2^. Figure 4 shows the engineering stress-train curves of the original sample (a hydrogen-uncharged sample) and samples with hydrogen-pre-charged. As shown in Figure 4a, the original sample achieved a plasticity of 27.33% with a yield strength of 465 MPa and an ultimate tensile strength of 585 MPa. After hydrogen charging, the presence of hydrogen markedly reduced the plasticity of the samples. The specific elongation and *I_HE_* of the samples under different hydrogen charging times are displayed in Figure 4b. Comparatively, 27Cr−4Mo−2Ni SFSS exhibited significant hydrogen embrittlement sensitivity compared to conventional ferritic stainless steel [14,26]. Increasing the hydrogen charging time from 0 min to 15 min resulted in significant changes in elongation and *I_HE_*. The elongation decreased from 27.33% to 7.98%, while the *I_HE_* increased from 0 to 71.00%. When the hydrogen charging time continued to increase, it was found that the elongation and *I_HE_* of the samples changed slightly, which were basically the same as those of the samples charged with hydrogen for 15 min. The elongation and hydrogen embrittlement susceptibility index *I_HE_* of the specimens were measured at 7.66% and 72.00%, respectively, which may be related to the hydrogen content in the hydrogen-charged samples. Figure 5 illustrates the variation in the *I_HE_* of the 27Cr−4Mo−2Ni SFSS concerning hydrogen content. These findings indicated that the maximum plastic damage occurred when the 27Cr−4Mo−2Ni SFSS samples were charged with hydrogen at a current density of 50 mA/cm^2^ for 15 min. Once the hydrogen content in 27Cr−4Mo−2Ni SFSS reached or surpassed 2.11 ppm, the plasticity loss induced by hydrogen saturation resulted in no further alterations.

**Figure 6:**
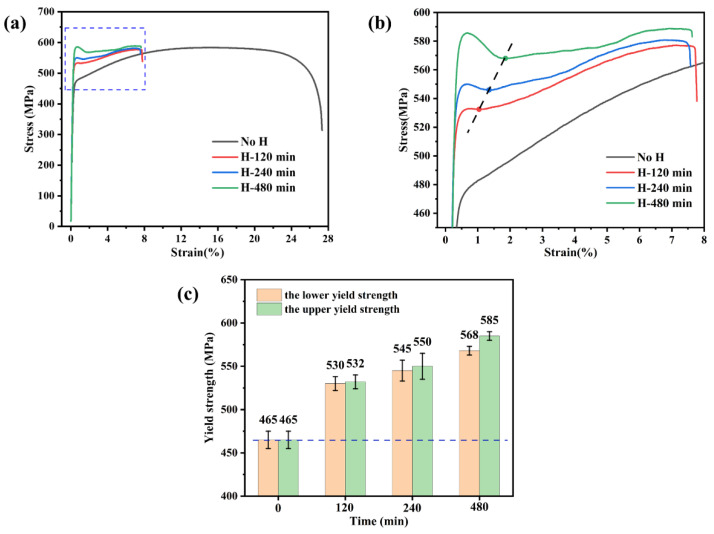
(**a**) The engineering stress-train curves of samples with different hydrogen-charged times (0, 120, 240, and 480 min); (**b**) the enlarged view of the blue box in (**a**) and (**c**) histograms of the upper and lower yield strengths versus hydrogen charging times.

However, the impact on the yield strength of the 27Cr−4Mo−2Ni SFSS specimens charged with hydrogen for varying times was notably significant. It can be observed from Figure 6a,b that a distinct yield phenomenon was observable in the stress-strain curves after long-term hydrogen charging. When the first positive peak (the upper yield point) is followed by a negative peak (the lower yield point), conventionally, the yield strength is identified as the stress at the first negative peak (the lower yield strength). The yield strength of the hydrogen-charged samples increased with the increase in hydrogen charge time. When the hydrogen charging time was short (from 5 to 15 min), the yield strength increased but not significantly, as shown in Figure 4a. However, with a longer hydrogen charging time, both the upper and lower yield strengths showcased substantial increments, i.e., from 465 MPa to 568 MPa for the lower yield strength and from 465 MPa to 585 MPa for the upper yield strength, as shown in Figure 6c. Notably, the difference between the upper and lower yield strengths expanded as the hydrogen charging time increased. Figure 6a,b illustrate an apparent drop in the flow stresses after reaching the upper yield point, and this drop became more significant with increasing hydrogen charging time of the samples. The stress-strain curve gradually flattened during the subsequent plastic deformation, and the work hardening became weaker.

### 3.3. Microstructural Characteristics

**Figure 7:**
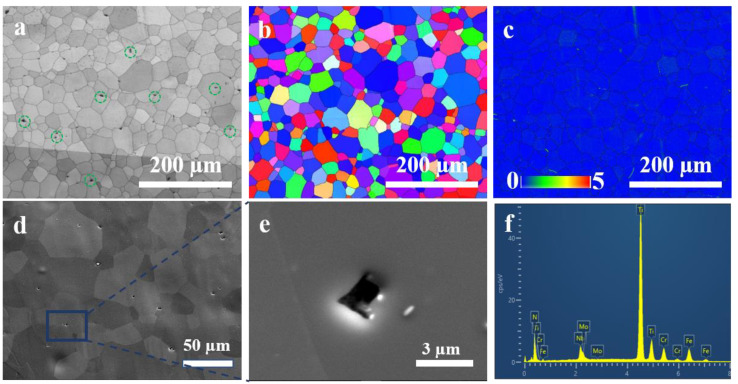
Microstructure of the 27Cr−4Mo−2Ni SFSS original sample: (**a**) the band contrast image, TiN particles are marked in the green dashed circle; (**b**) the inverse pole figure image; (**c**) the kernel average misorientation image; (**d**,**e**) the backscattered electron image; and (**f**) the energy dispersive spectroscopy image.

Figure 7 shows the microstructural characterization results of the investigated original sample of 27Cr−4Mo−2Ni SFSS. The band slope image and inverse pole figure image in Figure 7a,b presented the microstructure of the original sample, consisting of ferritic equiaxed grains with an average grain diameter of 22.62 µm. Some small cubic particles were observed randomly within the interior of ferritic grains and along grain boundaries, as indicated by the green circle in Figure 7a. Figure 7d–f show the backscattered electron image and energy dispersive spectroscopy images of these particles. The energy dispersive spectroscopy result indicated that the black particles with regular shapes were TiN precipitates, with a size range of 3–5 µm. Additionally, the kernel average misorientation image in Figure 7c represents the difference in the misorientation angle between the test point and the surrounding test points in the sample. This image reflected the degree of deformation, showing that the deformation in the original sample had disappeared.

### 3.4. Fractographic Observations

**Figure 8:**
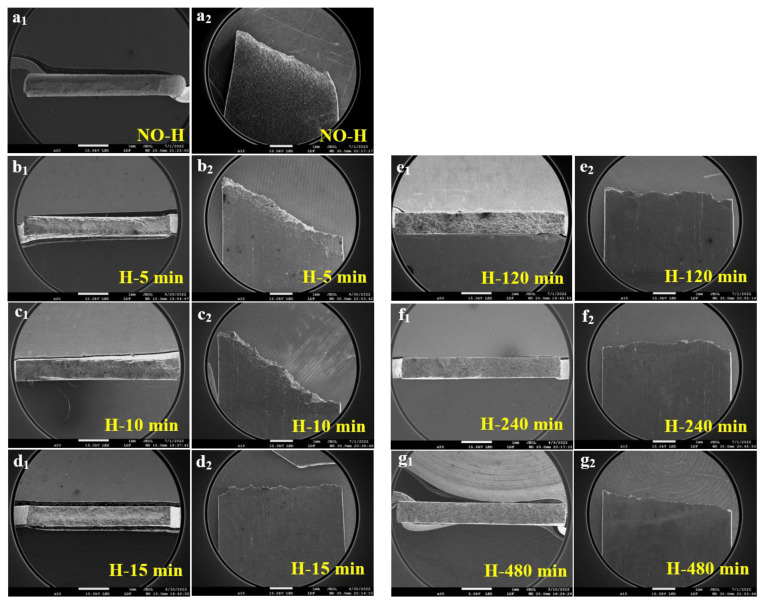
Low-magnification SEM images of the fracture morphology of samples that were hydrogen charged for different time at 50 mA/cm^2^: (**a_1_**,**a_2_**) hydrogen-uncharged; (**b_1_**,**b_2_**) 5 min; (**c_1_**,**c_2_**) 10 min; (**d_1_**,**d_2_**) 15 min; (**e_1_**,**e_2_**) 120 min; (**f_1_**,**f_2_**) 240 min and (**g_1_**,**g_2_**) 480 min. Subscript 1 presents the top view; subscript 2 shows the side views (RD × TD).

Figure 8 presents the low-magnification SEM images depicting the fracture morphology of the 27Cr−4Mo−2Ni SFSS samples subjected to varying hydrogen charging durations. Subscript 1 presents the top views; subscript 2 shows the side views (RD × TD). Figure 8(a_1_) demonstrates that the fracture of the hydrogen-uncharged sample exhibited an obvious necking phenomenon, characteristic of a typical ductile fracture. The micromorphology of the hydrogen-uncharged sample displayed a characteristic shear fracture pattern (Figure 8(a_2_)), with the fracture extending at an angle of approximately 45°. In contrast, the fracture surfaces of the hydrogen-pre-charged samples exhibited divergent morphologies depending on the hydrogen pre-charge time or the hydrogen concentration. (I) For short-term hydrogen charging (ranging from 5 min to 15 min), the edge zone displayed characteristics of brittle fractures while the central zone exhibited traits of ductile fractures. This resulted from the decrease in hydrogen content from the surface towards the interior of the sample. The proportion of the central ductile region decreased as the hydrogen charging time increased (Figure 8(b_1_,c_1_,d_1_)). Cracks emerged on the outer surface of the sample’s necking region, oriented perpendicular to the loading direction, as shown in Figure 8(b_2_,c_2_). The formation of these cracks was attributed to fractures within the embrittled layer at the boundary throughout the tensile process. (II) For long-term hydrogen charging (ranging from 120 min to 480 min), fractures observed in the hydrogen-charged samples displayed purely brittle fracture characteristics, with the fracture appearing smoother as hydrogen charging time increased (Figure 8(e_1_,f_1_,g_1_)). Macroscopically, the necking phenomenon was significantly reduced in the samples after hydrogen charging. Furthermore, with an increase in the hydrogen pre-charge time, the fracture mode transitioned from a shear fracture to a normal fracture and from a mixed ductile and brittle fracture to a solely brittle fracture.

**Figure 9:**
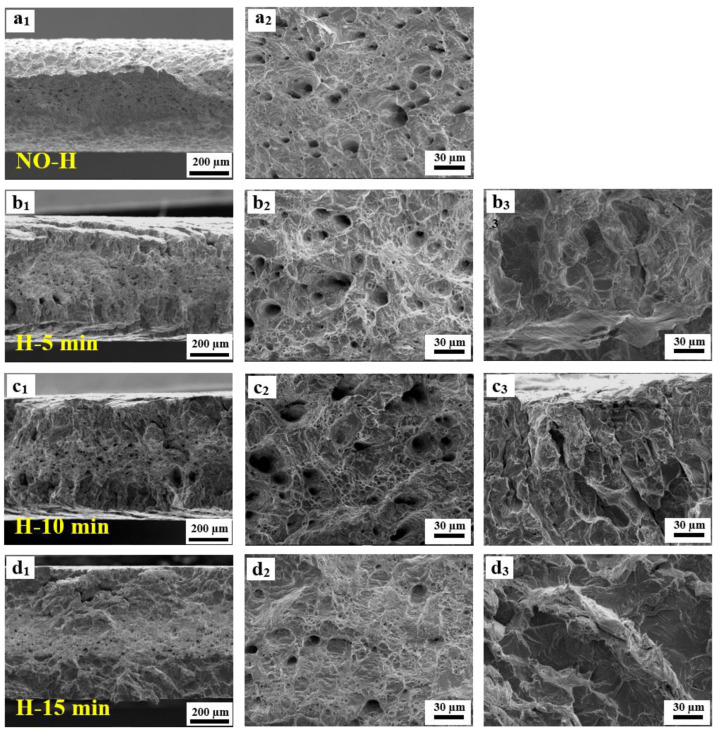
High-magnification SEM images of the fracture surfaces after short-term hydrogen charging. (**a_1_**,**b_1_**,**c_1_**,**d_1_**): Macroscopic fracture morphology. (**a_2_**,**b_2_**,**c_2_**,**d_2_**): Ductile region at the center of the samples. (**b_3_**,**c_3_**,**d_3_**): Brittle region at the edge in hydrogen-charged samples.

**Figure 10:**
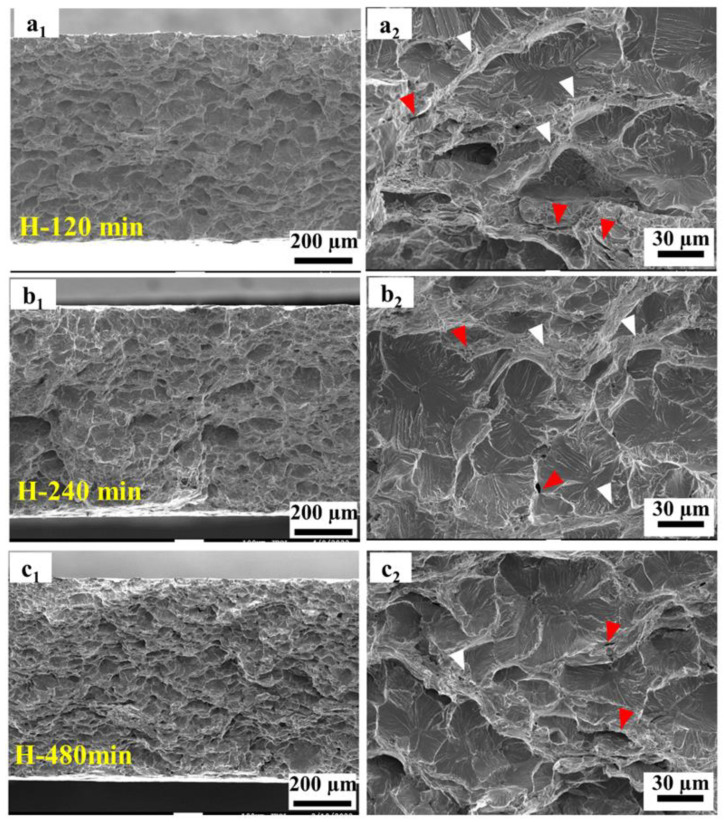
High-magnification SEM images of a hydrogen-charged sample under different times: (**a_1_**,**a_2_**): 120 min; (**b_1_**,**b_2_**): 240 min; and (**c_1_**,**c_2_**): 480 min. The red arrow indicates the secondary crack, and the white arrow indicates fine ductile voids.

Figure 9 and Figure 10 are high-magnification SEM images of the fracture surfaces. The entire fracture surface of the hydrogen-uncharged sample was characterized by ductile pores and dimples, as shown in Figure 9(a_1_,a_2_). The fracture surface of the tensile test samples charged with hydrogen exhibited a more complicated fracture behavior. Figure 9 shows the fracture morphologies for different electrochemical hydrogen charging times, i.e., 5, 10, and 15 min, respectively. The fracture exhibited a mixed failure mode of ductility and brittleness. The central ductile region was characterized by pores and dimples, as indicated in Figure 9(b_2_,c_2_,d_2_). As hydrogen pre-charge time increased, the number of pores decreased, the central ductile region decreased, and the thickness of the edge embrittlement layer gradually increased.

When the hydrogen charging time was 120 min or more, the sample fracture showed a complete brittle fracture mode. A “river-type” pattern can be observed on the fracture surface, as shown in Figure 10. The brittle fracture morphologies showed more complex states, including quasi-cleavage zones, secondary cracks, and a small number of fine ductile voids. The quasi-cleavage fractures originated from defects and traversed multiple neighboring grains. The extent of the cleavage extension zone decreased with increasing hydrogen content. At the convergence point between the two quasi-cleavage propagation zones, scarcely any fine dimples were noticeable.

**Figure 11:**
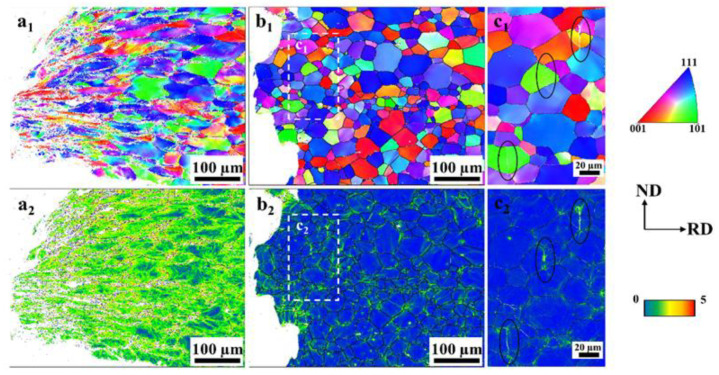
EBSD microstructure of the fracture surface. (**a_1_**,**a_2_**) Sample without hydrogen charged; (**b_1_**,**b_2_**) sample with hydrogen charged for 240 min; (**c_1_**,**c_2_**) the enlarged view of the white box in (**b_1_**,**b_2_**), the circle indicates the location of cracks. (**a_1_**,**b_1_**,**c_1_**) Inverse pole figure images (IPF); (**a_2_**,**b_2_**,**c_2_**) kernel average misorientation images (KAM).

Figure 11 shows the typical EBSD images of the cross-section near the fracture surfaces of the samples after tensile fracture without hydrogen charge and pre-charged with hydrogen for 240 min. Under hydrogen-uncharged conditions, the microstructure of the samples underwent plastic deformation only along the loading direction due to dislocation slip, as shown in Figure 11(a_1_,a_2_). The EBSD image of the side fracture surface of the hydrogen-pre-charged sample (in Figure 11(b_1_)) exhibited the transgranular fracture characteristic of hydrogen-induced crack propagation in 27Cr−4Mo−2Ni SFSS. Microcracks that formed during mechanical testing were observed in the grains, as shown in Figure 11(c_1_,c_2_). The KAM image reveals the distribution of strain. The five color tones employed in the KAM image represent five grades of the average misorientation angle between pixels (from 0° to 5°). A change from blue to red indicates a strain increase. The KAM images (Figure 11(b_2_,c_2_)) of the samples showed that electrochemical hydrogen charging resulted in strain localization in 27Cr−4Mo−2Ni SFSS, and the grain morphology of the fracture was basically unchanged. The deformation was localized mainly in the vicinity of the cracks and at the grain boundary, indicating that hydrogen facilitated dislocation motion in localized regions under applied stress.

## 4. Discussion

### 4.1. Hydrogen Trapping Sites

**Figure 12:**
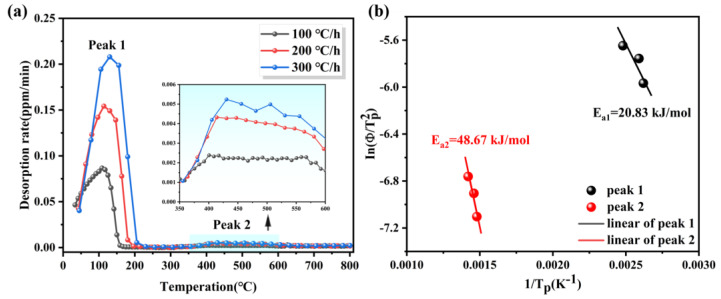
(**a**) Hydrogen thermal desorption spectra of 27Cr−4Mo−2Ni SFSS at different heating rates for 240 min. (**b**) Kissinger plots of $ln(\emptyset /T_{P}^{2})$ and $1/T_{P}$ for hydrogen desorption peak.

There are many locations where hydrogen can be trapped in stainless steels, including dislocations [27], grain boundaries [28], phase interfaces [26], and interfaces of inclusions or precipitates [29,30], and so on. To ascertain the hydrogen trapping locations within 27Cr−4Mo−2Ni SFSS, TDS was employed to obtain the hydrogen desorption activation energy ($E_{a}$) of the samples (with hydrogen-pre-charged samples for 240 min at 50 mA/cm^2^). This analysis involved tracking the shift in the hydrogen desorption peak under varying heating rates (100, 200, and 300 °C/h). The value of *E_a_* can be estimated from the model proposed by Kissinger (obtained from the following Kissinger first-order kinetics equation [31,32] (2)):

(2) $$\frac{\partial ln(\emptyset /T_{P}^{2})}{\partial (1/T_{P})}=-\frac{E_{a}}{R}$$

where Φ is the heating rate, K·h^−1^; $T_{P}$ is the desorption peak temperature, K; and *R* is the gas constant, 8.314 J·mol^−1^·K^−1^. Figure 12a presents the hydrogen thermal desorption spectra of 27Cr−4Mo−2Ni SFSS under varying heating rates. It is evident that with the increase in the heating rate, the position of the desorption peak shifted, and the temperature of the desorption peak gradually increased. Figure 12b shows the relationship between $ln(\emptyset /T_{P}^{2})$ and $1/T_{P}$ for the samples that underwent a hydrogen charge for 240 min at 50 mA/cm^2^. From this data, *E_a_* was estimated based on the slope of the straight line. According to the fitted linear curve in Figure 12b, the activation energies associated with the dual peaks were computed to be 20.83 kJ/mol and 48.67 kJ/mol, respectively. This estimated activation energy of peak 1 was also consistent with the value reported by other papers [33,34] regarding hydrogen dissociation from the grain boundary or dislocation diffusion. Combined with the kernel average misorientation diagram shown in Figure 7c, peak 1 was induced by the grain boundary. Peak 2, in particular, is linked to the release of hydrogen from the interfaces of inclusions or precipitates possessing high activation energy (48.67 kJ/mol). Combining with the backscattered electron image depicted in Figure 7d, peak 2 was induced by TiN particles. Peak 2 was induced by TiN particles. The hydrogen traps in 27Cr−4Mo−2Ni SFSS are predominantly located within lattices or grain boundaries, as well as TiN particles.

### 4.2. Fracture Behavior

**Figure 13:**
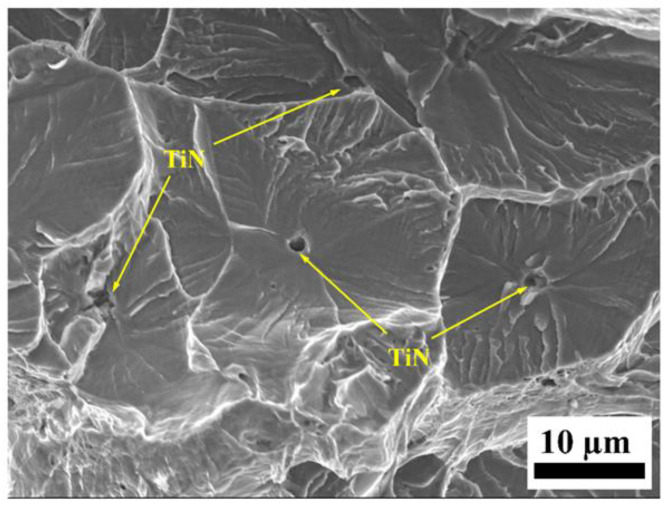
Typical SEM image of a hydrogen-charged sample under 480 min.

**Figure 14:**
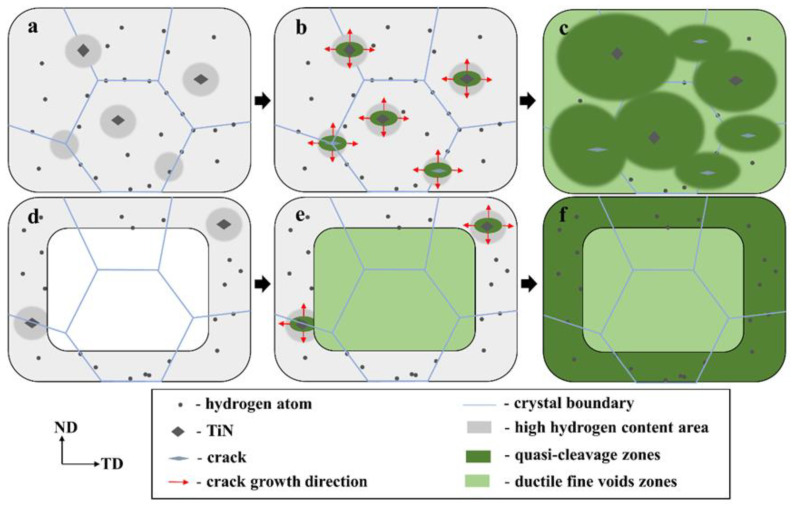
Schematic illustration of the fracture mechanism of 27Cr−4Mo−2Ni SFSS with hydrogen pre-charging for different times. (**a**–**c**) 120 min, 240 min, and 720 min; and (**d**–**f**) 5 min, 10 min, and 15 min.

In this study, we found that the diffusible hydrogen in the SFSS was concentrated more in the trapping sites with low activation energy. During the process of pre-charging hydrogen, the diffusible hydrogen was distributed throughout the microstructure. However, a high concentration of hydrogen was accumulated around the TiN and grain boundaries, as evidenced in Figure 12a. TiN particles have a higher Ea value compared to lattices or grain boundaries, indicating that TiN particles possess a greater propensity for capturing hydrogen atoms. Hydrogen accumulation in the vicinity of TiN will cause the hydrogen content to reach a critical level. Thus, the captured hydrogen atoms reduced the cohesive strength of the interface between TiN and the matrix due to the action of HDED. TiN became the main initiation point for cracks, as illustrated in Figure 13. The cracks then propagated in the radial direction from the initiation points, leading to the formation of round-shaped areas on the quasi-cleavage fracture surface, as shown in Figure 10 and Figure 13. The local strain occurred at the crack tip (Figure 11(c_2_)), and the newly generated dislocation acted as reversible traps for hydrogen. Hydrogen was supplied to the crack tip region through diffusion to maintain the high concentration of hydrogen atoms at the crack tip and promote crack growth in quasi-cleavage mode. Until the cracks met, a large number of dislocations gathered in the hydrogen-rich region at the crack tip, and high-density dislocations met and intersected with each other, forming a large number of vacancies, and eventually the crack grew in ductile microvoid coalescence mode (Figure 14c). As indicated by the white arrows in Figure 10(b_2_), ductile fracture occurred between the two quasi-cleavage zones. With the increase in hydrogen pre-charge time, the hydrogen concentration in the matrix not only increased, but also the hydrogen content in the vicinity of the TiN and grain boundaries reached the critical value. The number of crack initiation points increased and the extent of the crack extension zone decreased with increasing hydrogen content, as shown in Figure 10.

During the hydrogen charging process, hydrogen atoms permeate from the surface to the core of the specimen. When hydrogen was pre-charged for a short time, only hydrogen was distributed on the sample surface (Figure 14d). Therefore, the ductile void zone can be observed in the middle, and the quasi-cleavage area can be observed at the edge of the specimen fracture (Figure 14e,f). When the hydrogen charging time was 15 min, the thickness of the hydrogen diffusion layer reached the critical thickness of the normal fracture of the sample. Additionally, the hydrogen content approached a critical threshold, resulting in the peak of hydrogen-induced plastic damage in 27Cr−4Mo−2Ni SFSS.

### 4.3. Effect of Hydrogen on Mechanical Properties

As can be seen from the tensile curve in Figure 4a, hydrogen significantly diminished the plasticity of SFSS, with the degree of plastic loss escalating alongside the increase in hydrogen charging time (i.e., the increase in hydrogen content), aligning with the findings of numerous research studies [14]. However, as shown in Figure 4a, the effect of hydrogen on strength in 27Cr−4Mo−2Ni SFSS differed significantly. In this study, after hydrogen was pre-charged, the ultimate tensile strength of the 27Cr−4Mo−2Ni SFSS did not change much, and the yield strength of the material had a significant increase. The little change in ultimate tensile strength was attributed to hydrogen-induced premature material failure. The yield strength increased with the increase in hydrogen content, the fundamental reason for which was the pinning effect of hydrogen on dislocations [35]. The significant lattice distortion surrounding dislocations played a key role, acting as an attractive trap. Hydrogen atoms accumulated toward the dislocation because they were sensitive to strain fields. Hydrogen atoms were captured by the physical trap, i.e., the dislocation core [36]. Dislocation core atoms had a higher energy compared to the rest due to the lattice mismatch. Studies [37] have shown that the H atoms occupy the dislocation core interstitial position, reducing the lattice mismatch at the edge dislocation core. Hence, H atoms reduced the dislocation core energy in BCC Fe and increased the shear stress necessary for dislocation mobilization. As a result, the yield strength of the samples that were pre-charged with hydrogen for a short time increased. In addition, a large number of diffused hydrogen atoms accumulated at the dislocation core after the long-time hydrogen pre-charging, forming a hydrogen atom air mass, the so-called Cottrell atmosphere. It is generally believed that due to the large diffusion coefficient of hydrogen atoms, the gas atmosphere can move with the dislocation at room temperature. However, the diffusion coefficient of hydrogen was related to the hydrogen concentration according to the following formula [38] (3):

(3) $$D^{*}=D(1+\frac{\partial \ln\gamma }{\partial \ln c})$$

where *D* is the diffusion coefficient at low hydrogen concentrations; $D^{*}$ is the diffusion coefficient at higher hydrogen concentrations; γ is the activity coefficient; and c is the hydrogen concentration. When the concentration of hydrogen in the material was high, the interaction between hydrogen atoms decreased γ, and the hydrogen diffusion coefficient also decreased. Hence, the Cottrell atmosphere had a pinning effect on the dislocations, which hindered the dislocation movement in 27Cr−4Mo−2Ni SFSS. During the tensile deformation process, dislocations move under the pinning effect of the Cottrell atmosphere, which requires additional stress. This phenomenon contributed to the relatively high yield strength of the material [39]. As shown in Figure 6, there was an apparent yield platform on the stress-strain curve after long-time hydrogen pre-charging. The disparity between the upper and lower yield strengths progressively widens as the hydrogen charging time is prolonged. This trend indicated an increasingly pronounced pinning effect of the Cottrell atmosphere as hydrogen concentrations increased. And the longer the hydrogen charge time, the higher the required additional stress and the higher the upper and lower yield strengths.

Furthermore, once the plastic deformation stage was initiated, the high stress environment within the tensile specimen led to dislocation and high slip velocity. At the same time, due to the effect of HEDE, the diffused hydrogen reduced the cohesive strength of the crystal lattice and decreased the resistance to dislocation motion, i.e., the Peierls–Nabarro stress. Under the effect of both, the dislocations move rapidly in the crystal. Dislocation entanglement was reduced, and the work-hardening rate decreased. This effect becomes more significant in samples with high hydrogen concentrations.

## 5. Conclusions

The effects of hydrogen on the mechanical properties and the fracture behavior of 27Cr−4Mo−2Ni super ferritic stainless steel were investigated through the electrochemical cathode hydrogen charging experiment with different hydrogen charge times. The main findings are as follows:

1. The 27Cr−4Mo−2Ni SFSS exhibits a significant hydrogen-embrittlement sensitivity. The elongation decreased significantly with the introduction of hydrogen. When the sample was charged with hydrogen for 15 min, the elongation decreased from 27.33% to 7.98%. Nevertheless, once the elongation reaches a critical value (7.98%), further reductions are minimal even with additional hydrogen introduction due to the saturation of plastic deformation loss caused by hydrogen.
2. The yield strength of the SFSS exhibits a monotonic increase with hydrogen content due to the formation of the Cottrell atmosphere. The hydrogen-charged SFSS shows an obvious drop in flow stress after upper yielding, followed by a weak work-hardening deformation stage. The more hydrogen is charged, the more the flow stress drops and the lower the work hardening rate becomes.
3. The fracture morphology of samples subjected to hydrogen charging is contingent upon the time of hydrogen charging and the hydrogen content. An extension of the hydrogen pre-charge time induces a transition in fracture behavior from a combination of ductile and brittle features to a predominantly brittle quasi-cleavage fracture. Concurrently, there is a discernible shift in the fracture mode from a shear fracture to a normal fracture.

## Figures and Tables

**Table 1:** The chemical composition of 27Cr−4Mo−2Ni SFSS (wt%).

C	Cr	Mn	Mo	Nb	Ni	Ti	Si	Cu	N	P	S	Fe
0.015	27.57	0.23	3.72	0.37	1.98	0.14	0.4	0.05	0.016	0.022	0.002	Bal.

## Data Availability

Data are contained within the article.
